# Strain‐Adaptive Dielectric Metamaterials via Bioinspired “Ligament‐Bone” Architecture for Ultrahigh‐Energy Capacitive Storage

**DOI:** 10.1002/advs.76253

**Published:** 2026-06-24

**Authors:** Jian Wang, Xinyu Wang, Jiabao Wang, Biyun Peng, Yifei Zhang, Ning Zhu, Xin Hu

**Affiliations:** ^1^ Ningxia Key Laboratory of Photovoltaic Materials School of Materials and New Energy Ningxia University Yinchuan China; ^2^ College of Materials Science and Engineering Jiangsu National Synergetic Innovation Center for Advanced Materials Nanjing Tech University Nanjing China; ^3^ College of Biotechnology and Pharmaceutical Engineering State Key Laboratory of Materials‐Oriented Chemical Engineering Nanjing Tech University Nanjing Jiangsu China; ^4^ Chemistry Department University of Alberta Edmonton Canada

**Keywords:** Al_2_O_3_@BaTiO_3_, carrier traps, dielectrics, energy storage, ligament‐bone bionics

## Abstract

Polymer dielectrics for capacitive energy storage face fundamental trade‐offs between breakdown strength, energy density, efficiency, and mechanical robustness. Herein, we break this paradigm by designing a bioinspired strain‐adaptive dielectric metamaterial with a multiscale “ligament‐bone” architecture. The “ligament” phase epoxy‐functionalized polyvinylidene fluoride‐based polymer provides dynamic constraints to suppress ferroelectric loss, while the “bone” units, alumina‐coated barium titanate nanocores (Al_2_O_3_@BaTiO_3_), engineered with a strain‐responsive “periosteum” shell, mitigate interfacial distortion and carrier migration. This hierarchical design synergistically enables unprecedented electro‐mechanical properties: a record‐high energy density of 26.1 J cm^−3^ with 90.2% efficiency at 600 MV m^−1^, coupled with a Young's modulus of 2.13 GPa. Operando characterizations and multiscale simulations reveal that strain‐adaptive reconfiguration of polymer chains and core‐shell interfaces dynamically optimizes field/charge distribution under extreme conditions. This biomimetic strategy establishes a universal framework for designing next‐generation dielectrics for extreme‐condition electronics.

## Graphical abstract

A bioinspired strain‐adaptive ligament‐bone architecture achieves record‐high energy density of 26.1 J cm^−3^ and 90% efficiency at 600 MV m^−1^, coupled with a Young's modulus of 2.13 GPa.

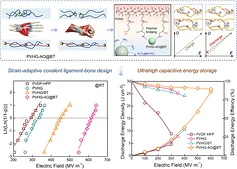

## 1 Introduction

The urgent demand for compact, efficient energy storage in electric vehicles, grid technologies, and pulsed power systems has propelled polymer film capacitors to the forefront of dielectric research. While the commercial benchmark biaxially oriented polypropylene (BOPP) exhibits low loss, its low polarizability and poor mechanical toughness limit the achievable energy density to merely ∼2 J cm^−3^ at 600 MV m^−1^, restricting next‐generation applications [1, 2, 3]. Ferroelectric polymers like polyvinylidene fluoride (PVDF) and its copolymers offer higher permittivity, yet their energy storage is fundamentally limited by ferroelectric hysteresis, low breakdown strength (*E*_b_< 400 MV m^−1^), and intrinsic brittleness as illustrated in Figure S1 [4, 5, 6]. Despite decades of efforts incorporating blending, crosslinking, and nanofiller engineering, the critical “trilemma” of achieving ultrahigh energy density (*U*_d_ >25 J cm^−3^) [7, 8, 9], efficiency (>90%), and mechanical robustness remains unresolved. Invariably, enhancements in one property compromise others.

In biological systems, hierarchical architectures leverage dynamic strain adaptation to achieve exceptional mechanical resilience. For instance, the tendon‐bone junction, comprising soft collagen fibers interwoven with rigid mineralized phases, exhibits graded stiffness that redistributes stress concentrations under sudden loading. Concurrently, the periosteum actively remodels microcracks through strain‐responsive cellular signaling [10, 11, 12, 13]. Translating such dynamic load‐management mechanisms to dielectric composites could resolve the persistent electro‐mechanical trade‐offs [14, 15, 16]. Recent studies have explored bioinspired static designs: soft‐hard polymer matrices (e.g., core‐shell PVDF‐based copolymers) mitigate ferroelectric loss, while rigid‐core/soft‐shell fillers (e.g., SiO_2_@BaTiO_3_, Al_2_O_3_@CCTO) decouple polarization from breakdown [17, 18, 19]. Yet, these approaches remain fundamentally static and cannot emulate nature's real‐time reconfiguration capability. Under high electric fields (>500 MV m^−^^1^), dynamic electro‐mechanical coupling becomes dominant: interfacial charge injection induces lattice distortion at filler‐matrix boundaries, electromechanical stress waves propagate microcracks, and carrier avalanche triggers thermal runaway [20, 21, 22, 23]. Current static biomimetic designs fail to address these transient phenomena, leading to irreversible damage and rapid performance decay, a critical bottleneck for industrial applications.

**FIGURE 1:**
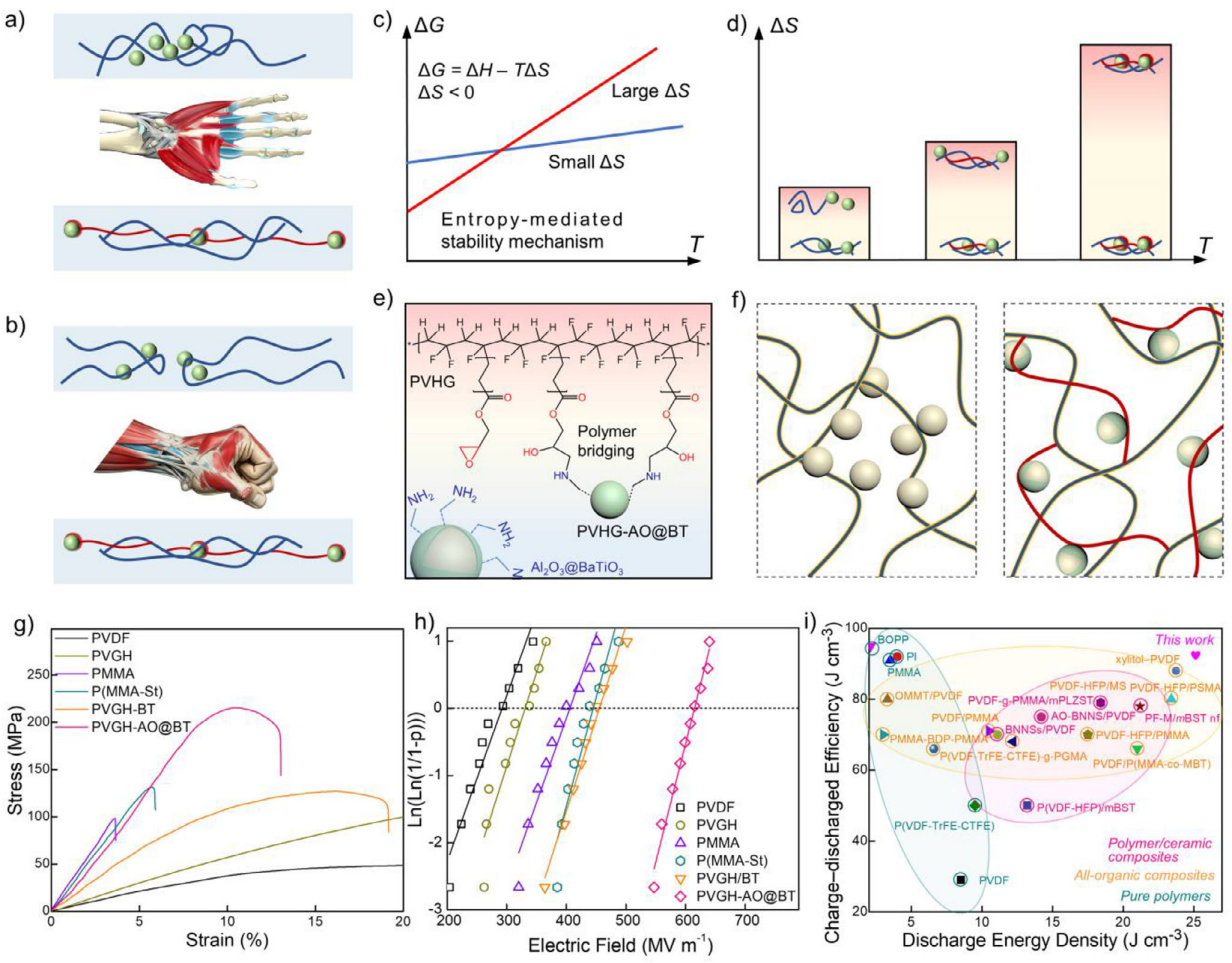
(a, b) Schematic diagram of biomechanical strain in ligament‐bone systems. (c, d) Mechanism of molecular ligaments reducing Δ*S* and enhancing mechanical stability in composites. (e, f) PVHG ring‐opening epoxy‐based anchoring mechanism of AO@BT and reconstruction of nano/molecular structures. (g) Stress‐strain curve and (h) Weibull distribution of *E*_b_ for film dielectrics. (i) Comparison of *U*_d_ and *η* for film dielectrics in recent years.

Here, we bridge this gap with a strain‐adaptive dielectric metamaterial inspired by the “ligament‐bone” biomechanics (Figure 1a,b) [24, 25, 26]. We engineer (i) PVDF‐HFP‐g‐PGMA (PVHG) molecular ligaments that covalently anchor to fillers via epoxy‐amine reactions, suppressing ferroelectric relaxation and enhancing mechanical integrity, and (ii) BaTiO_3_ nanocores coated with an amino‐functionalized Al_2_O_3_ periosteum shell (AO@BT) that provides stress buffering and deep‐trap carrier immobilization (Figure 1c,d) [27, 28, 29]. Crucially, both components exhibit field‐induced strain adaptability, reconfiguring their nano/molecular structures to dissipate local stresses and redistribute *E*‐fields dynamically (Figure 1e,f; Figures S2 and S3) [30, 31]. This bioinspired strategy culminates in an unprecedented combination of properties: a *E*_b_ of 625 MV m^−1^ (133% higher than pristine PVDF), an energy density of 26.1 J cm^−3^ (600% enhancement), 90.2% efficiency, and a Young's modulus of 2.13 GPa (Figure 1g,h; Figure S4, Table S1), surpassing most reported polymer dielectrics (Figure 1i) [1, 2, 3, 4, 5, 6, 7, 32, 33, 34, 35, 36, 37]. Combined operando spectroscopy, in situ scattering, and multiscale modeling decode the synergy of ligand constraint, periosteum deformation, and trap‐modulated charge blocking, establishing a universal blueprint for dielectrics under extreme electromechanical conditions, opening avenues for resilient energy storage in flexible electronics, aerospace systems, and beyond.

## 2 Results and Discussion

**FIGURE 2:**
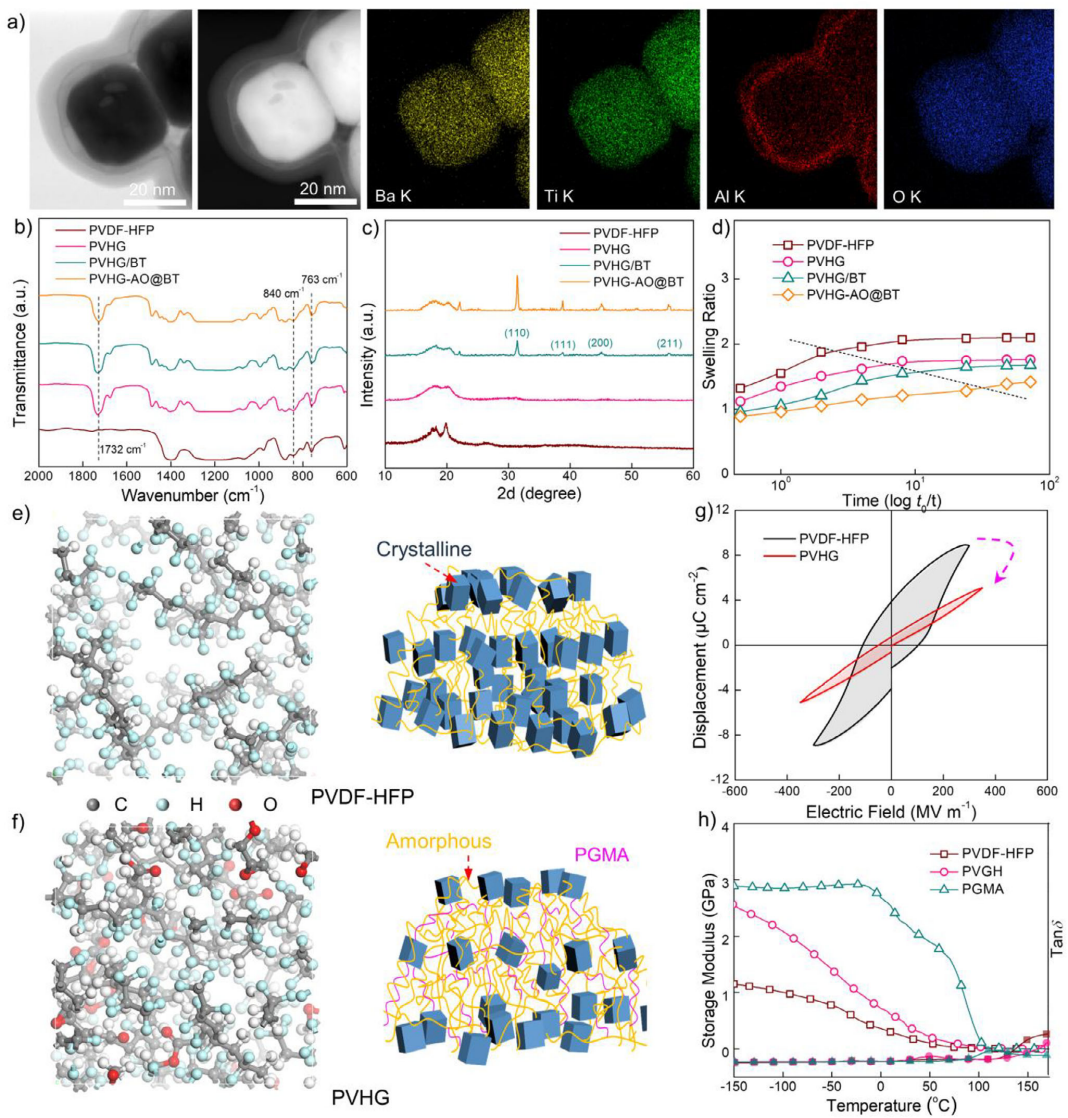
(a) HRTEM and its elemental distribution of AO@BT. (b) FT IR, (c) XRD, (d) Swelling characteristics of PVDF‐HFP, PVHG, PVHG/BT, PVHG‐AO@BT films. (e) PVDF‐HFP, (f) PGMA molecular packing arrangements and schematic diagrams of crystalline/amorphous phases. (g, h) Polymer hysteresis loops and DMA curves.

TEM analysis (Figure 2a) reveals a stable core‐shell architecture for the AO@BT nanoparticles, characterized by uniform morphology and a distinct Al_2_O_3_ layer, with elemental mapping confirming the homogeneous distribution of all components. The structural evolution from semi‐crystalline PVDF‐HFP with mixed *α*/*β* phases to a deliberately disordered state after GMA grafting underscores our design strategy. FTIR (bands at 763/840 cm^−1^) and XRD (reflections at 18.5°/20.3°) confirm the initial phases (Figure 2b,c). Grafting linear PGMA chains reduces crystallite size and overall crystallinity from 42% to 28% (DSC) and introduces critical ester carbonyl motifs (C═O stretch at 1732 cm^−1^) and pendant epoxy groups [2, 3]. The carbonyls engage in dipole‐dipole interactions with the PVDF matrix while the epoxide rings covalently react with the amino‐functionalized Al_2_O_3_ shell surface, forming molecular ligaments that chemically tether the matrix to the rigid fillers and create an interface capable of dissipating strain energy. This “ligament‐bone” architecture achieves remarkable mechanical stabilization, as evidenced by solvent swelling kinetics in *N*‐methyl‐2‐pyrrolidone (Figure 2d). The PVHG‐AO@BT composite exhibits a significantly lower equilibrium swelling ratio (*SR* = 1.15) than the PVHG/BT blend (*SR* = 1.52), alongside a twelvefold increase in the diffusion equilibrium time (48 h vs. 4 h). This stark contrast underscores the formation of a multi‐anchored network in which PGMA ligaments entangle with PVDF‐HFP chains via dipole‐dipole interactions while covalently grafting to the Al_2_O_3_ shell (Figure S5) via epoxy‐amine reactions, thereby constructing a gradient interface that dissipates strain energy and restricts chain mobility [4, 5]. The inclusion of BaTiO_3_ cores (distinct XRD peaks at 31.5° (101) and 45.3° (200)) further enhances polarization density [9, 35], while the deliberately introduced oxygen vacancy gradients within the Al_2_O_3_ interlayer create deep traps that suppress charge injection.

The strategic incorporation of PGMA as molecular ligaments fundamentally transforms the ferroelectric response of PVDF‐HFP. The confined crystallization process reduces ferroelectric domain sizes [5, 6, 7], resulting in significant suppression of both the magnitude and temporal evolution of ferroelectric relaxation. The complete miscibility between PGMA side chains and the PVDF matrix, coupled with specific dipole‐dipole interactions between ester carbonyls (C═O) and C─F bonds (Figure 2e,f; Figure S6), promotes dense chain packing and further dilutes the crystallinity [5, 26], an effect more pronounced in grafted architectures than in blends. Functioning as dynamic molecular struts, the PGMA segments subdivide macroscopic ferroelectric domains, reducing remanent polarization (*P*_r_) from 7.2 µC cm^−2^ to 2.5 µC cm^−2^, and undergo reversible field‐induced conformational changes, as directly evidenced by operando FTIR spectroscopy, which confine polarization reversal and enable rapid switching kinetics synchronized with external field variations (Figure 2g; Figure S7). This collective action effectively converts the high‐loss ferroelectric PVDF into a low‐loss relaxor‐ferroelectric [5, 32], characterized by a slim hysteresis loop and a 68% reduction in energy loss (Figure S8), thereby establishing the essential foundation for high‐efficiency energy storage. Although homopolymer PGMA is brittle with a high modulus (∼3.0 GPa), its role as a molecular tether covalently anchored to the AO@BT nanoparticles via epoxy‐amine reactions bridges the soft PVDF matrix and rigid fillers, yielding a synergistic enhancement in mechanical properties. A notable increase in the DMA storage modulus is observed across all temperatures (Figure 2h), and the fracture energy rises to 5.8 kJ m^−2^, representing a threefold increase over the unmodified system.

**FIGURE 3:**
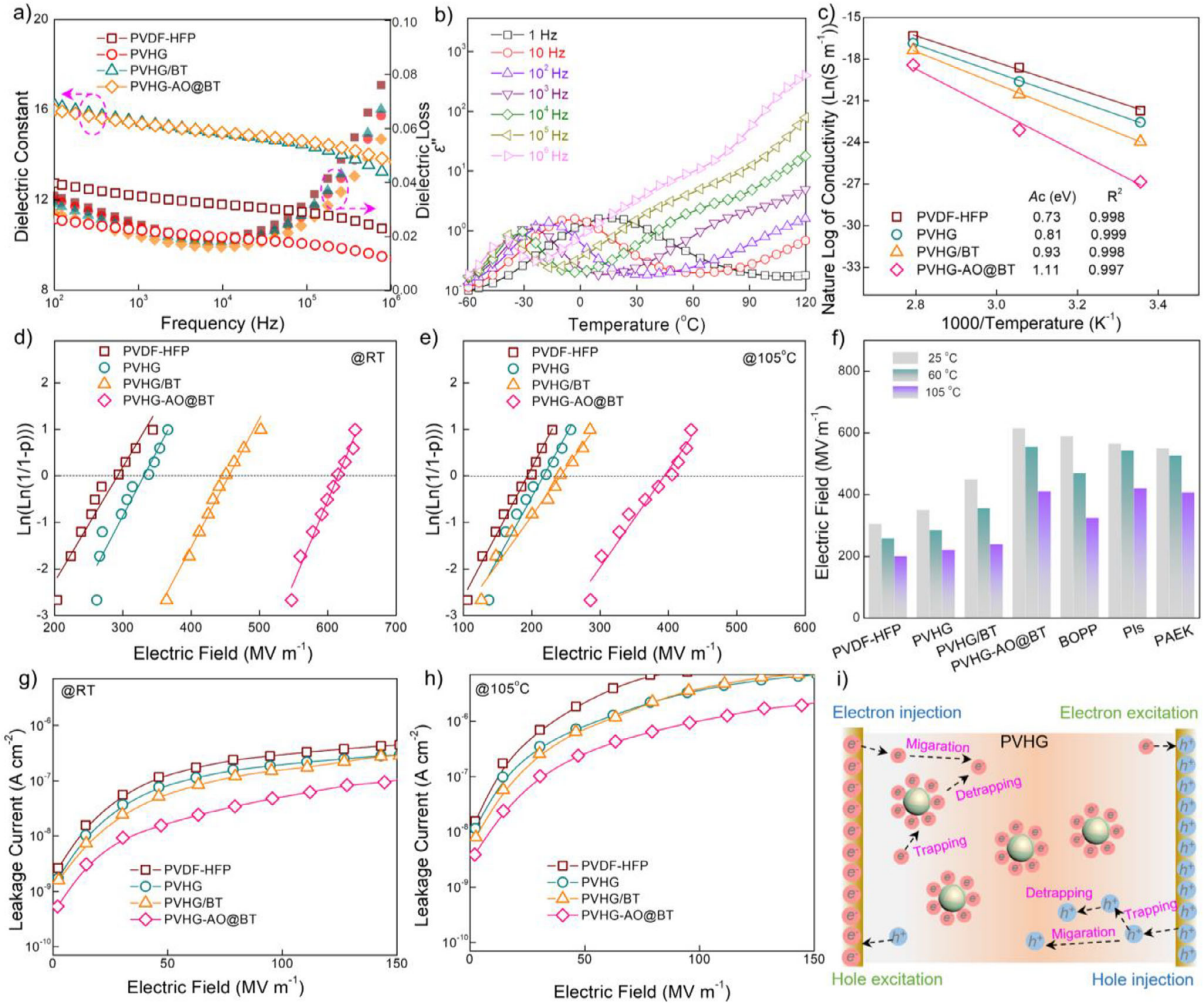
(a) Dielectric constant and dielectric loss of PVDF‐based polymer films. (b) Dielectric temperature spectrum of PVHG‐AO@BT films. (c) The relationship between the conductivity of the films and temperature, along with a comparison of their activation energy. (d) 25°C, and (e) 105°C; (f) comparison of *E*_b_ of polymer dielectric films at different temperatures. Electric field‐dependent conduction current density at (g) 25°C and (h) 105°C under difference *E*‐field of polymer dielectrics. (i) Schematic of the bipolar carrier transport model in composite films.

The multifunctional role of molecular ligaments is prominently manifested in the dielectric response of the polymer composites. As shown in Figure 3a, the low‐dielectric‐constant PGMA dilutes the overall polarity of PVDF‐HFP and disrupts the cooperative response of ferroelectric domains, leading to a reduced dielectric constant (from 12.9 to 10.4 at 1 kHz) but a concurrently lower dielectric loss (tan*δ* from 0.027 to 0.025). Introducing a small amount of high‐permittivity BaTiO_3_ (*ε*_r_ ∼2000) significantly enhances the dielectric constant of the PVHG/BT composite to 15.6, albeit with an increased tan*δ*. Critically, the epoxy‐amine‐bonded Al_2_O_3_ interlayer mitigates tanδ to 0.022 across the full temperature range (Figure 3b), confirming that covalent bridging effectively suppresses interfacial loss between the low‐εr matrix and high‐εr filler [9]. The significantly suppressed electrical conductivity from room to elevated temperature correlates with the enhanced insulating properties of the composite film. This is directly supported by the increased activation energy (*A*_c_) calculated from the Arrhenius plot in Figure 3c and Figure S9. The dielectric *E*_b_ governed by synergistic electrical, thermal and mechanical failure mechanisms, was statistically analyzed using a two‐parameter Weibull distribution (Figure 3d,e; Figure S10). The *E*_b_ of pristine PVDF is approximately 290 MV m^−1^ at room temperature. The PGMA segments, confirmed by operando FTIR to reconfigure dynamically under field, enhance *E*_b_ to 321 MV m^−1^ by constraining domain switching damage. Nano‐sized BT particles, acting as charge scattering centers, further increase *E*_b_ to 448 MV m^−1^, although excessive loading causes a sharp decline due to interfacial defects and field distortion. The strategic intercalation of an amino‐functionalized layer, covalently bonded to the PGMA matrix, introduces deep interfacial traps that boost the *E*_b_ of PVHG‐AO@BT to 625 MV m^−1^. Remarkably, this composite maintains a high *E*_b_ of 403 MV m^−1^ at 105°C, the upper operational limit for commercial BOPP, significantly surpassing BOPP and rivaling intrinsic high‐temperature polymers like PEI and PI, while retaining the high polarity inherent to PVDF‐based systems. Furthermore, thickness‐dependent measurements across 5 to 20 µm films confirm robust dielectric performance with a Weibull shape parameter *β* exceeding 20 and *E*_b_ remaining above 590 MV m^−1^ even at 20 µm in Figure S11. Leakage current measurements (Figure 3g,h) and subsequent analysis based on the *J‐E* conduction model reveal a substantially increased charge hopping distance (*λ*) in PVHG‐AO@BT films at both room and elevated temperatures, accounting for the superior insulation. Figure 3i and Figure S12 illustrate that the improved *E*_b_ originates from interfacial carrier trapping, which lowers the local space charge density and causes a subsequent suppression of the leakage current.

**FIGURE 4:**
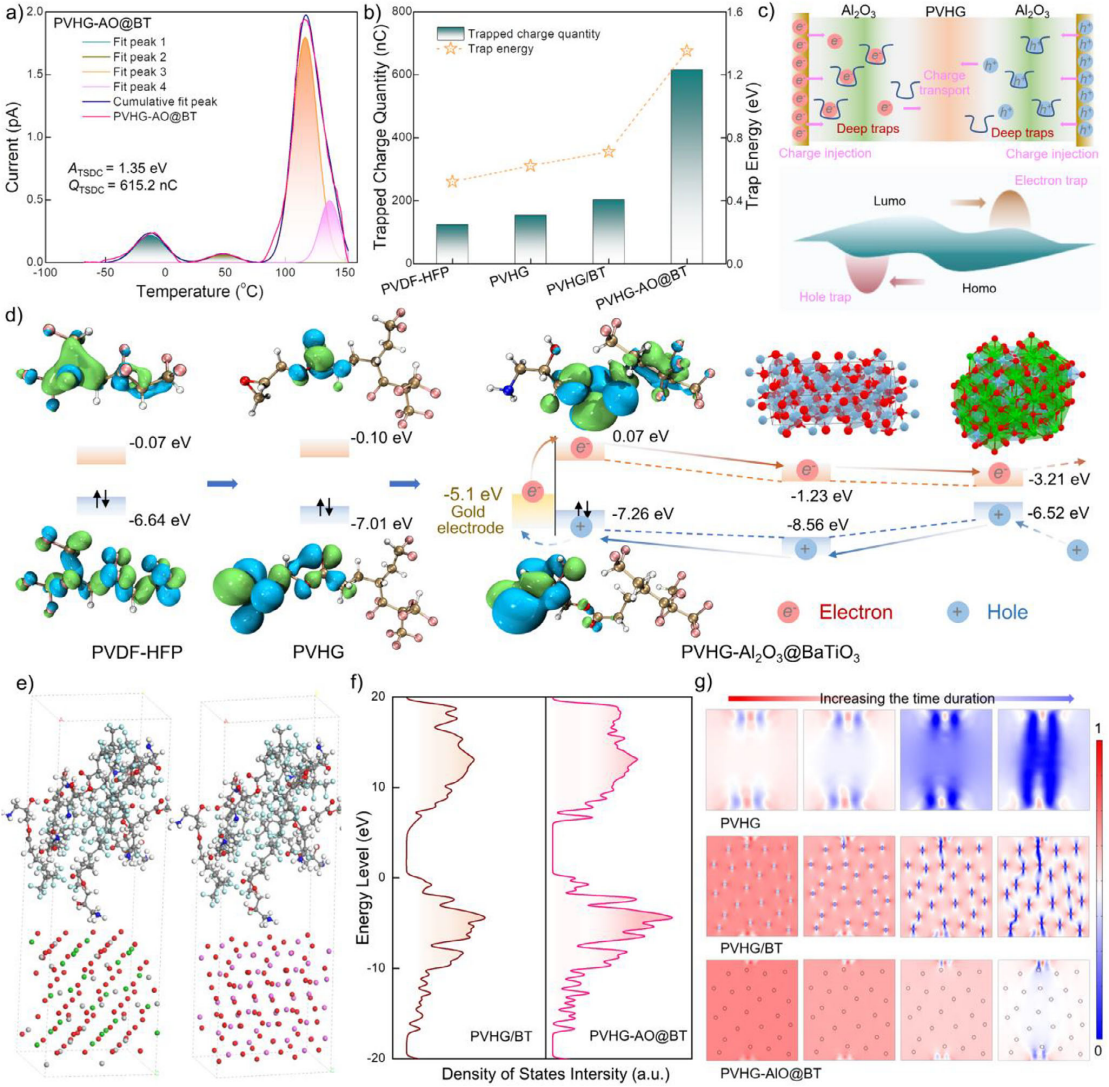
(a) The TSDC curves of (a) PVHG‐AO@BT nanocomposites, and (b) the *Q*_TSDC_ and *A*_TSDC_ of polymer‐based films calculated from TSDC curves. (c) Schematic illustration of the influence of the Al_2_O_3_ shell layer on charge transport and its interfacial traps. (d) Band structure and electron‐hole transport mechanisms in polymers, fillers, and their nanocomposites. PVHG/BT and PVHG‐AO@BT (e) molecular stacking structures and (f) their density of states. (g) Phase‐phase simulation analysis of electrical breakdown paths in different dielectric films.

The enhancement of energy density in dielectric polymers, being proportional to *E*_b_^2^, necessitates a mechanistic understanding of how the covalently anchored molecular ligament architecture suppresses charge transport. Thermally stimulated depolarization current (TSDC) analysis, deconvoluted using the partial heating method (Figure 4a; Figure S13), reveals that the PVHG‐AO@BT exhibits not only a characteristic peak associated with the glass transition near −25 °C but also a dominant current peak with a larger integrated area and a significantly higher depolarization temperature. This is accompanied by a minor peak attributable to interfacial bonding within PVHG‐AO@BT, collectively indicating the introduction of deeper trap levels (1.24 eV, as quantified in Figure 4b) compared to pure PVDF or PVHG (Figure 4c), which effectively immobilizes space charges (trapped charge: 601 nC). DFT calculations provide atomic‐level insight into this charge‐blocking mechanism.

Electronic structure analysis (Figure 4d; Figure S14) reveals that the incorporation of PGMA and its subsequent ring‐opening reaction with amino groups widen the bandgap of the polymer matrix and shift both the conduction band minimum (CBM) and valence band maximum (VBM) (Figure S15) [15, 16]. The electrostatic potential further confirms an enriched charge interface in the modified polymer, enhancing charge adsorption. The wide‐bandgap Al_2_O_3_ interlayer, covalently integrated via the epoxy‐amine linkage, forms a substantial interfacial energy barrier between the polymer matrix and the BaTiO_3_ core [19, 28]. This design not only directly impedes carrier injection from the high‐permittivity filler but also establishes a dual‐trap system that suppresses bidirectional electron‐hole transport. Concurrently, at the film‐electrode interface, the elevated conduction band edge of the modified polymer forms a higher Schottky barrier, significantly reducing charge injection from the electrodes. Consequently, this multi‐scale trap engineering strategy, combining deepened bulk traps with the enhanced interfacial barrier established by the dynamic covalent linkage, minimizes both electrode injection and bulk transport losses [7, 38], resulting in the exceptionally low leakage current observed in PVHG‐AO@BT films, as graphically summarized in the charge transport schematic.

A mesoscale computational analysis of the PVHG/BT and PVHG‐AO@BT composites, based on their distinct polymer‐filler spatial distributions (Figure 4e), reveals a significantly widened effective bandgap and a characteristic deep‐trap density of states in the PVHG‐AO@BT system (Figure 4f). This electronic structure modification, arising from the covalent epoxy‐amine bonding between the PGMA ligands and the amino‐functionalized Al_2_O_3_ shell, provides a robust energy barrier that suppresses charge injection and impedes carrier transport, consistent with our DFT calculations and the enhanced activation energy measured by TSDC. To fundamentally decipher the rapid breakdown process, we performed deterministic simulations combining a fractal dielectric breakdown model with a percolation theory using MATLAB and COMSOL Multiphysics 5.4b in Figure 4g and Figure S16 [6, 7]. The simulations demonstrate that the dielectric constant gradient across the covalently bonded PGMA/Al_2_O_3_/BaTiO_3_ interface, coupled with the dynamic conformational response of the molecular ligaments evidenced by operando FTIR (Figure S17) and in situ SAXS (Figure S18), causes substantial redistribution of the electric potential that deflects breakdown paths. In stark contrast to the rapid and extensive dendritic propagation observed in pristine PVDF, electrical tree growth in the PVHG‐AO@BT films is profoundly suppressed. The developing branches are initially blocked by the BaTiO_3_ nanoparticles, increasing the path tortuosity, and are further confined by the wide‐bandgap, highly insulating Al_2_O_3_ shells. This confinement leads to an increased Debye length due to potential loss along the evolving tree and traps the charge carriers at the multiple PVHG/AO@BT interfaces [39, 40]. Consequently, the initiation and propagation of electrical trees in PVHG‐AO@BT are significantly retarded, yielding dispersed and constrained branches that prevent early‐stage damage and suppress the complete breakdown pathway, thus accounting for the ultrahigh *E*_b_.

**FIGURE 5:**
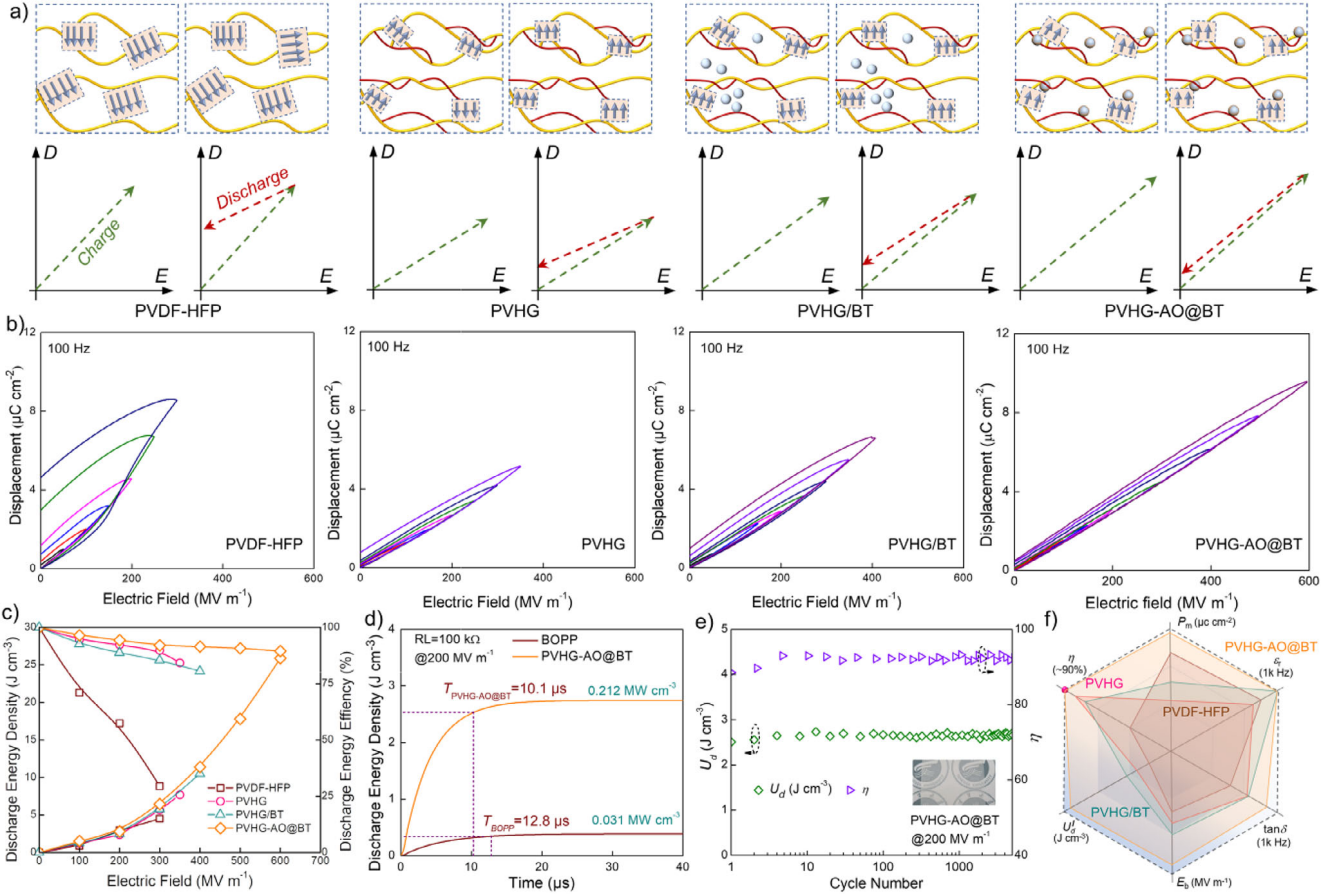
(a) Schematic illustration demonstrating the aggregated state structure and polarization‐depolarization of PVDF‐based polymers. (b) the *D‐E* loops and (c) *U*_d_ and *η* of dielectric films. (d) Discharge rate and (e) charge‐discharge cycle stability of PVHG‐AO@BT nanocomposite and BOPP films. (f) Comparison of the comprehensive electrical properties of dielectric films.

Beyond the critical parameter of *E*_b_, the polarization characteristics of a dielectric fundamentally govern its energy storage performance. The maximum polarization (*P*_m_) dictates the upper limit of energy density (*U*_d_), while the remanent polarization (*P*_r_), though often overlooked in high‐permittivity polymers, directly determines the dischargeable *U*_d_ and thus the operational efficiency (*η*). Low *η* leads to significant heat generation during charge‐discharge cycles, posing serious risks of thermal runaway and device failure in Figure 5a. Therefore, enhancing energy storage necessitates a concurrent achievement of high *P*_m_ and minimized *P*_r_. To ensure measurement accuracy, we employed large‐area electrodes (5 mm diameter) for the *D‐E* loop characterization, as control experiments confirmed that the *ε*_r_ (∼20) is independent of electrode size, thereby mitigating the parasitic capacitance and fringe effects prevalent in small‐electrode configurations that artificially inflate both *ε*_r_ and *E*_b_. As designed, the PGMA molecular ligands, covalently anchored to the AO@BT fillers via epoxy‐amine reactions, resulting in a substantially reduced *P*_r_ from 7.2 µC cm^−2^ to 2.5 µC cm^−2^. The introduction of high‐*ε*_r_ BaTiO_3_ nanoparticles further refines the PVDF crystallinity and significantly boosts the overall *P*_m_. Most importantly, the synergistic architecture featuring AO@BT nanofillers covalently tethered via epoxy‐amine linkages to the PGMA ligands not only preserves the high *P*_m_ but also introduces deep interfacial traps and dynamic stress buffering at the ligament‐bone interface. This unique design enables simultaneously high *P*_m_ and exceptionally low *P*_r_ compared to the other three films, as unambiguously evidenced by the slim, saturated *D‐E* loops measured at 100 Hz (Figure 5b; Figure S19). Consequently, the combination of a high *P*_m_‐*P*_r_ value and a superior *E*_b_, reinforced by a *Y* of 2.13 GPa that mechanically suppresses electromechanical breakdown, forms the cornerstone for achieving high *U*_d_ coupled with high *η*, a critical advancement for reliable, high‐power dielectric capacitors.

The energy storage performance, quantified by integrating the *D‐E* loops, demonstrates the transformative impact of the molecular ligament architecture. The PVHG‐AO@BT nanocomposite film achieves an exceptional *U*_d_ of 26.1 J cm^−3^ at 600 MV m^−1^, as shown in Figure 5c, representing a 600% improvement over the pristine PVDF‐HFP film and a 1300% enhancement compared to commercial BOPP. Crucially, this ultrahigh energy density is coupled with a remarkably high *η* of 90%, a benchmark rarely attained in conventional PVDF‐based polymer dielectrics and a fundamental prerequisite for practical applications. The charge–discharge dynamics, characterized by the time required to reach 90% of the maximum energy density (Figure 5d), reveal an ultrafast charging time of 10.1 µs for the PVHG‐AO@BT films, outperforming BOPP (12.8 µs). Furthermore, the nanocomposite exhibits outstanding operational reliability, retaining over 99% of its initial *U*_d_ and *η* after 5000 cycles at 200 MV m^−1^ (Figure 5e) and maintaining stable performance with less than 5% fluctuation after 2000 cycles at 500 MV m^−1^ (Figure S20), demonstrating exceptional fatigue resistance that originates from the dynamic strain dissipation enabled by the epoxy‐amine tethered PGMA ligands. In addition, we confirmed the spatial uniformity, thickness‐insensitive breakdown (Figure S21), and cross‐filler generality of the capacitive performance. The consistent energy density and efficiency were maintained across the film, and this performance is also successfully extended to alternative filler systems (Figure S22). The overall superiority of the dielectrics contextualized within a comprehensive performance map of dielectric polymers (Figure 5f, Ashby plot) It unambiguously validates that the biomimetic ligament‐bone strategysuccessfully orchestrates the soft matrix and rigid fillers. By constructing covalently bridged and dynamically adaptive interface, this strategy yields a combination of high energy density, high efficiency, rapid discharge, and excellent endurance.

## 3 Conclusion

In conclusion, we demonstrate a bioinspired strain‐adaptive dielectric metamaterial that overcomes the long‐standing electro‐mechanical trade‐offs in polymer capacitors. By mimicking the “ligament‐bone” architecture, we design a dynamic system where molecular “ligaments” suppress ferroelectric loss, while “bone” units with a responsive “periosteum” shell mitigate interfacial distortion and carrier migration. This hierarchical design enables an unprecedented combination of an ultrahigh energy density of 26.1 J cm^−3^ and 90.2% efficiency at 600 MV  m^−1^, together with a Young's modulus of 2.13 GPa. Operando studies and simulations reveal that strain‐adaptive reconfiguration optimizes field/charge distribution under extreme conditions. This biomimetic approach establishes a universal framework for designing next‐generation dielectrics for high‐power and flexible electronics.

## 4 Experimental Section

### 4.1 Materials

Barium titanate nanoparticles (BaTiO_3_, 100 nm, 99%, Aladdin). Aluminium sulfate octadecahydrate (99.5%, Macklin) (≥98%), Ammonium formate (99%, Aladdin), formic acid (99.5%, Macklin), glycidyl methacrylate (GMA, 98%, Aladdin), and 10‐phenylphenothiazine (98%, Rhawn), Poly(vinylidene fluoride‐*co*‐hexafluoropropylene)(PVDF‐HFP, Arkema, Kynar Flex 2801, VDF:HFP = 90:10). *N*,*N*‐dimethylformamide(DMF, 99.5%, Sinopharm)and anhydrous ethanol were dried over molecular sieves before use. Other chemicals or solvents were purchased from Shanghai Aladdin Industrial Inc. and used as received without further purification.

### 4.2 Synthesis of Al_2_O_3_‐Coated BaTiO_3_ (AO@BT) Nanoparticles

Ammonium formate was dissolved in deionized water and stirred until completely dissolved. Formic acid was added to adjust the pH to 4.6, yielding a buffer that regulates the precipitation kinetics of the alumina precursor while also serving as a latent ammonia source for surface functionalization. Then, BaTiO_3_ nanoparticles and aluminum sulfate octadecahydrate were added to this buffered solution. The resulting mixture was ultrasonically dispersed for 30 min, followed by heating in a water bath at 70°C for 2 h. Afterward, the white solid particles were centrifuged and washed several times with deionized water until the pH approached neutral. Finally, the resultant was placed in a ceramic crucible and heated in a muffle furnace at 500°C for 2 h to obtain the BaTiO_3_@Al_2_O_3_ particles with a continuous amorphous Al_2_O_3_ shell of ∼5 nm thickness, as verified by high‐resolution TEM and XPS analysis.

### 4.3 Preparation of PVHG‐AO@BT Composite Films

The PVHG copolymer was synthesized via organo‐catalyzed atom transfer radical polymerization (O‐ATRP) to avoid any metal residue. PVDF‐HFP was dissolved in DMSO, followed by the addition of 10‐phenylphenothiazine (10‐PTH) under N_2_ atmosphere. After adding glycidyl methacrylate, the reaction was proceeded under the irradiation of UV light (365 nm) at room temperature with a fan for cooling the environment temperature. The product was precipitated in the mixture of methanol and water, washed with methanol several times, extracted by CHCl_3_ for 12 h to remove the homopolymer PGMA, and dried under vacuum at 30°C overnight. Successful grafting was confirmed by FTIR spectroscopy, showing characteristic epoxy and carbonyl stretches. The PVHG copolymer and the amino‐functionalized AO@BT nanoparticles were co‐dispersed in DMF through sequential ultrasonication and mechanical stirring. The homogeneous suspension was blade‐coated onto clean glass substrates using a programmable film applicator. The films underwent stepwise drying: 60°C for 6 h, 80°C for 2 h, and final annealing at 120°C for 1 h to promote epoxy‐amine crosslinking between the PGMA grafts and amino‐functionalized filler surfaces. Film thickness was precisely controlled at 10 ± 1 µm using a digital micrometer. The cross‐linked composite films exhibited excellent mechanical integrity and flexibility, suitable for subsequent electrical characterization.

### 4.4 Characterization

Crystal structures were analyzed by X‐ray diffraction (XRD, Rigaku) with Cu Kα radiation over a 2θ range of 10°–50°. Chemical states were examined via X‐ray photoelectron spectroscopy (XPS) on an AXIS SUPRA+ instrument. Molecular vibrations and functional groups were characterized by Fourier‐transform infrared (FTIR) spectroscopy in ATR mode (Nicolet iS50), accumulating 32 scans at 4 cm^−1^ resolutions. Operando FTIR measurements under applied electric fields were conducted using a custom‐built sample cell with ITO‐coated transparent electrodes, allowing spectral acquisition while the DC bias was ramped from 0 MV m^−1^ to 500 MV m^−1^. In situ small‐angle X‐ray scattering (SAXS) under DC electric fields was performed at a synchrotron beamline (BL19U2, SSRF) using a custom‐designed high‐voltage sample stage. The morphological features and the cross‐sectional structure were examined by field‐emission scanning electron microscopy (FE‐SEM, Hitachi SU8020, Japan) and transmission electron microscopy (HRTEM, JEM‐200CX, Japan). Dielectric properties were evaluated with a Novocontrol Concept‐80 impedance analyzer under an AC voltage of 1 V across 30–250°C. Ferroelectric hysteresis (*D‐E* loops) and leakage current density were measured using a Huace2000 ferroelectric tester. Breakdown strength (*E*_b_) was determined via DC ramp tests with an automatic voltage withstand tester (DW‐P503‐1ACGFO). To determine the breakdown strength (*E*_b_) of dielectric polymers, at least 15 measurement points were selected from different regions of each sample. The *E*_b_ values along with the shape parameter *β* were derived from the two‐parameter Weibull distribution, as given by Equation (1):

(1) $$F\left(x\right)=1-\exp\left(-{\left(\frac{x}{\alpha }\right)}^{\beta }\right)$$

where *F*(*x*) represents the cumulative probability of electrical breakdown, *x* is the measured breakdown strength, *α* is the scale parameter, and *E*_b_ corresponds to the breakdown strength at a cumulative probability of 63.2% [7, 41, 42]. The shape parameter β reflects the scatter in the data; higher values of *β* indicate greater material reliability. Moreover, the enhanced *E*_b_ observed in polymer nanocomposites is associated with an increase in Young's modulus (*Y*), as described by the Stark‐Garton model in Equation (2):

(2) $$E_{b}=0.6\times \sqrt{\frac{Y}{\varepsilon_{0}\varepsilon_{r}}}$$

This model, widely applied to polymer‐based films, suggests that the improvement in mechanical toughness contributes to superior insulating performance. Previous studies have also shown that charge transport in dielectrics under high electric fields and elevated temperatures follows a jump conduction mechanism. Discharge energy density (*U*_d_) and efficiency (*η*) were calculated as Equations (3) and (4):

(3) $$U_{d}=\int EdD=\frac{1}{2}\varepsilon_{r}\varepsilon_{o}{E_{b}}^{2}$$

(4) $$\eta =\frac{U_{d}}{U_{d}+U_{l}}$$

where *ε*_0_ is the vacuum permittivity (8.85 × 10^−12^ F m^−1^) and *ε*_r_ is the relative permittivity, *η* was measured using a custom‐built charge‐discharge system [2, 3, 4]. Thermally stimulated depolarization currents (TSDC) were measured in conjunction with a 6517B ±10 kV high‐voltage amplifier, using a high‐voltage/temperature test system from RT to 200°C. TSDC data were analyzed using the half‐width method, and the trap energy levels were estimated according to Equation (5):

(5) $$A_{TSDC}=\frac{2.47\kappa T_{p}^{2}}{\Delta T}$$

where *T*_p_ represents the peak‐current temperature, Δ*T* is the half‐height of the peak. The quantity of trapped charge was calculated using the following in Equation (6):

(6) $$Q_{TSDC}=\left(\frac{60}{v_{0}}\right)\int_{t_{1}}^{t_{0}}I(T)dT$$

where *ν*_0_ is the heating rate (5 K min^−1^), t_0_ and t_1_ are the starting and ending temperatures of the peak, *I*(T) represent the TSDC curve.

Computational Methods: Density functional theory (DFT) calculations were performed using the Gaussian 16 package. The B3LYP hybrid functional with the 6–311G(*d*,*p*) basis set was employed to optimize molecular geometries of PVDF‐HFP repeating units and PGMA segments. This level of theory was chosen for its reliable description of dipole moments associated with C–F and C = O groups. Electronic properties, including frontier molecular orbital energies (HOMO/LUMO), electrostatic potential (ESP) distributions, and projected density of states (PDOS), were extracted from the optimized structures [43, 44]. ESP surfaces were mapped onto electron density isosurfaces of 0.002 a.u. and quantitatively analyzed using Multiwfn software to evaluate charge transfer characteristics at polymer‐filler interfaces, as further detailed in the Supporting Information.

All‐atom molecular dynamics (MD) simulations were conducted using LAMMPS with the COMPASS III force field, an ab initio‐based high‐quality force field capable of describing organic polymers, inorganic oxides, and their interfaces. Force field parameters for PVDF‐HFP and PGMA were obtained from the COMPASS III database. Nonbonded interactions between the polymer and the hydroxylated α‐Al_2_O_3_ (0001) surface were described using Lennard‐Jones potentials combined with atomic partial charges derived from our DFT calculations, following the Lorentz–Berthelot combination rules. Composite systems containing PVHG matrix and AO@BT nanoparticles were constructed with periodic boundary conditions. After energy minimization, systems were equilibrated in the NPT ensemble at 300 K and 1 atm for 2 ns, followed by production runs of 10 ns. Interfacial binding energies, chain orientation parameters [44, 45], and radial distribution functions were analyzed to quantify the strain‐adaptive behavior of the “ligament‐bone” architecture. Molecular configurations and dynamic evolution were visualized using VMD.

### 4.5 Phase Field Simulation

Electric field and stress distribution in the composite microstructure were analyzed using the AC/DC and Structural Mechanics modules. Representative volume elements were reconstructed based on experimental filler distribution, and coupled electro‐mechanical simulations were performed to visualize the strain‐adaptive reconfiguration under high electric fields (>500 MV m^−^^1^). Multiphysics simulations of electrical breakdown were implemented in COMSOL Multiphysics 5.4b using a coupled phase‐field and electrostatic approach. The evolution of breakdown pathways was described by solving the time‐dependent Ginzburg‐Landau equation for the damage field variable φ, coupled with Poisson's equation for the electric potential distribution. The model incorporated experimentally determined material parameters for each phase, with particular attention to the dielectric constant mismatch at core‐shell interfaces. Breakdown initiation and propagation were simulated under increasing electric fields to elucidate the confinement effect of AO shells on dendritic tree growth [46, 47]. This behavior is represented by the *s*(*x*, *t*), as defined by Equations (7 and 8):

(7) $$\frac{\partial }{\partial \bar{x_{i}}}\left[\frac{1}{f(s)+\eta }.\frac{\partial \bar{\phi }}{\partial \bar{x_{i}}}\right]=0$$

(8) $$\frac{\partial s}{\partial \bar{t}}=f^{\prime }\left(s\right)+\frac{\partial^{2}s}{2\partial \bar{x_{i}}\partial \bar{x_{i}}}-\frac{f^{\prime }\left(s\right)}{2{\left[f(s)+\eta \right]}^{2}}.\frac{\partial \bar{\phi }}{\partial \bar{x_{i}}}.\frac{\partial \bar{\phi }}{\partial \bar{x_{i}}}$$

where the model employs the dimensionless form of the Euler equation along with the breakdown of *E*‐field variables. The breakdown path observed in the dielectric is represented as a dark blue growth path originating near the electrode.

## Author Contributions

**Yifei Zhang**: software, visualization. **Jian Wang**: data curation, investigation, formal analysis, visualization, writing – original draft, methodology, validation. **Biyun Peng**: data curation, formal analysis. **Jiabao Wang**: visualization, investigation. **Xin Hu**: writing – review and editing, conceptualization, funding acquisition, resources, supervision, formal analysis, project administration. **Ning Zhu**: conceptualization, supervision, funding acquisition, writing – review and editing. **Xinyu Wang**: methodology, validation, investigation, data curation, formal analysis, visualization, writing – original draft.

## Conflicts of Interest

The authors declare no conflict of interest.

## Supporting information




**Supporting File**: advs76253‐sup‐0001‐SuppMat.docx.

## Data Availability

The data that support the findings of this study are available from the corresponding author upon reasonable request.
