# Supplementary material for: Strain‐Adaptive Dielectric Metamaterials via Bioinspired “Ligament‐Bone” Architecture for Ultrahigh‐Energy Capacitive Storage
Source: Adv Sci (Weinh). 2026 Jun 24;13(52):e76253. doi: 10.1002/advs.76253 (PMC13336855; doi:10.1002/advs.76253)
Supplement: Supplementary file 1 [file ADVS-13-e76253-s001.docx]

**Support Information**

**Strain-Adaptive Dielectric Metamaterials via Bioinspired “Ligament-Bone” Architecture for Ultrahigh-Energy Capacitive Storage**

Jian Wang,^a^ Xinyu Wang,^b^ Jiabao Wang,^b^ Biyun Peng,^a^ Yifei Zhang,^d^ Ning Zhu,^c^* Xin Hu^b^*

^a^ *Ningxia Key Laboratory of Photovoltaic Materials, School of Materials and New Energy, Ningxia University, Yinchuan 750021, China*

^b^ *College of Materials Science and Engineering, Jiangsu National Synergetic Innovation Center for Advanced Materials,* *Nanjing Tech University,* *Nanjing 211800, China*

^c^ *College of Biotechnology and Pharmaceutical Engineering, State Key Laboratory of Materials-Oriented Chemical Engineering, Nanjing Tech University, Nanjing, Jiangsu 211800, China*

^d^ *Chemistry Department, University of Alberta, Edmonton T6G2N4, Canada*

Correspondence: ningzhu@njtech.edu.cn (N.Z.) and xinhu@njtech.edu.cn (X.H.)

The capacitors to store electrical energy is related to its polarization and an externally applied electric field (*E*). The energy storage density (*U_e_*), energy loss (*U_l_*) and charge-discharge efficiency (*η*) are expressed as: $U_{d}=\int_{P_{r}}^{P_{\mathrm{m}}} EdD$,$U_{l}=\int_{0}^{P_{r}} EdD$and $\eta=U_{d}/\left(U_{d}+U_{l}\right)$, where *P_max_* is the maximum polarization value and is positively correlated with the dielectric constant (*ε*_r_) of the polymer dielectrics as presented in **Fig. S1**. Obviously, high-energy storage performance dielectrics require high *P*_max_ and low *P_r_*.

**Fig. S1.** Schematic diagram and formula for calculating energy storage density and efficiency of dielectrics.

**Fig. S2.** Schematic of the “ligament-bone” structure of PVHG-AO@BT composites.

**Fig. S3.** Schematic diagram of the synthesis of polymer nanocomposites.

**Fig. S4.** Comparison of Young's modulus strength and breakdown field strength in polymer and composite films.

**Fig. S5.** The FT IR and XPS of AO@BT.

**Tab. S1**. Comparison of Force-electric data for polymer and composite films.

| Sample | Dielectric constant | Young’s modulus  (*Y,* GPa) | *E*_b_, theory,  MV m^-1^ | *E*_b_, experiment  MV m^-1^ |
| --- | --- | --- | --- | --- |
| PVDF-HFP | 12.9 | 0.42 | 1150 | 290.4 |
| PVHG | 10.4 | 0.64 | 1540 | 321.2 |
| PMMA | 3.9 | 3.12 | 4770 | 400.2 |
| P(MMA-St) | 3.9 | 2.78 | 4500 | 425.6 |
| PVHG-BT | 15.6 | 1.54 | 1680 | 448.3 |
| PVHG-AO@BT | 15.8 | 2.13 | 1960 | 624.9 |

**Fig. S6.** Molecular structural formulae for PVDF-HFP and PVHG.

**Fig. S7.** D-E loops of a) PVDF-HFP, b) PVHG.

**Fig. S8.** Polar structure of ferroelectric, relaxor ferroelectric, paraelectric, linear polymers, and a schematic diagram of their D-E loops.

**Fig. S9.** The electrical conductivity of polymer-based films at different temperatures and their comparison.

**Fig. S10.** Weibull distribution of the dielectric breakdown strength of thin films at 60 °C

**Fig. S11.** Weibull breakdown strength as a function of film thickness from 5 to 20 μm showing moderate field reduction.

**Fig. S12.** Schematic of charge transport in polymerized dielectric films under *E*-fields.

**Fig. S13.** The TSDC curves of PVDF films.

**Fig. S14.** Energy band structure of difference polymers.

**Fig. S15.** The HOMO and LUMO results of difference polymers.

**Fig. S16.** Phase-field simulation of electric field and leakage current in PVHG/BT and PVHG-AO@BT composite films.

**Fig. S17.** The in-situ electric field FT-IR of PVHG-AO@BT films.

**Fig. S18.** The in-situ electric field SXAS of PVHG-AO@BT films.

**Fig. S19.** Maximum polarization (*P*_m_) and remnant polarization (*P*_r_) of a) PVDF-HFP, b) PVHG, c) PVHG/BT, and d) PVHG-AO@BT dielectric films at the maximum E-field, e) along with their comparison.

**Fig. S20.** The charge-discharge cycle stability of PVHG-AO@BT nanocomposite films.

**Fig. S21.** The thickness mapping and regional energy storage performance across six different areas of PVHG-AO@BT nanocomposite films.

**Fig. S22.** Generality validation through breakdown strength comparison across different filler systems with and without covalent epoxy-amine bridging.
